# P-1759. Biomarkers and early sepsis: Comparative analysis of PSP, CRP, and Procalcitonin in the Dominican Republic

**DOI:** 10.1093/ofid/ofaf695.1930

**Published:** 2026-01-11

**Authors:** Yori A Roque, Alfredo J Mena Lora, David De Luna, Katherine M Cruz, Luz Maria Cruz, Roanny Mercedes Genao

**Affiliations:** Hospital Metropolitano de Santiago (HOMS), Santiago, Santiago, Dominican Republic; University of Illinois Chicago, Chicago, Illinois; Hospital Metropolitano de Santiago, Santiago, Santiago, Dominican Republic; Hospital Metropolitano de Santiago, Santiago, Santiago, Dominican Republic; Hospital Metropolitano de Santiago, Santiago, Santiago, Dominican Republic; Hospital Metropolitano de Santiago, Santiago, Santiago, Dominican Republic

## Abstract

**Background:**

Sepsis is a life-threatening condition caused by a dysregulated host response to infection, often leading to multiorgan failure and death. Mortality remains over 25%, making early diagnosis essential in critically ill patients. The need for reliable biomarkers readily available in low and middle income countries is of major interest. Pancreatic Stone Protein (PSP), produced in the pancreas and GI tract, has shown promise as a diagnostic and prognostic marker in sepsis, trauma, and inflammatory conditions. This study evaluated the role of PSP, procalcitonin (PCT), and C-reactive protein (CRP) as early predictors of sepsis in ICU patients at tertiary center in the Dominican Republic.

**Table 1.**
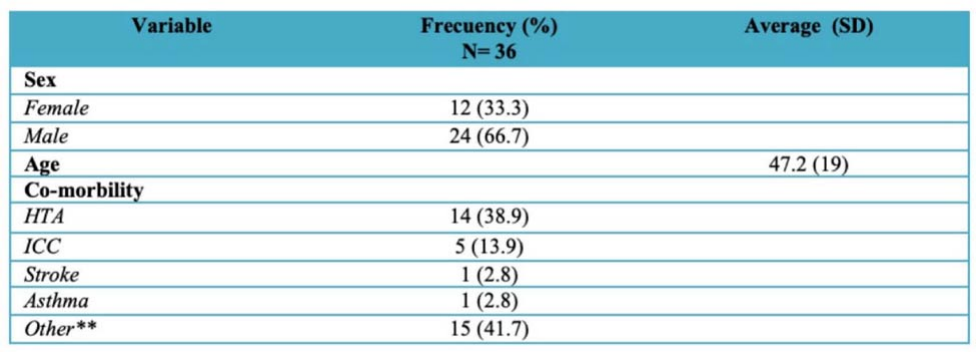
Demographic Factors and Comorbidities of Patients Admitted to the Intensive Care Unit (ICU) of the Hospital Metropolitano de Santiago (HOMS) During the Period September – December 2024

**Table 2.**
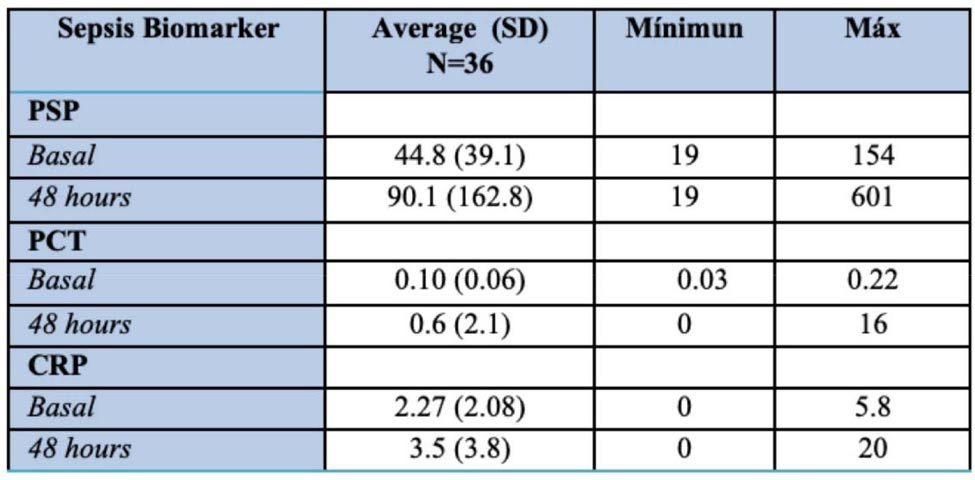
Results of Sepsis Biomarkers at Admission and During Follow-Up of Patients Admitted to the Intensive Care Unit (ICU) of the Hospital Metropolitano de Santiago (HOMS).

**Methods:**

This was a descriptive, observational, correlational study using secondary data from ICU patients at HOMS between September 1 and December 31, 2024. Of 343 screened records, 36 patients met inclusion criteria (no sepsis on admission). Data on demographics, biomarker levels (PSP, CRP, PCT), SOFA-based sepsis diagnosis, ICU length of stay (LOS), and mortality were analyzed using descriptive statistics, ANOVA, and Spearman correlation (p < 0.05).

**Figure 1.**
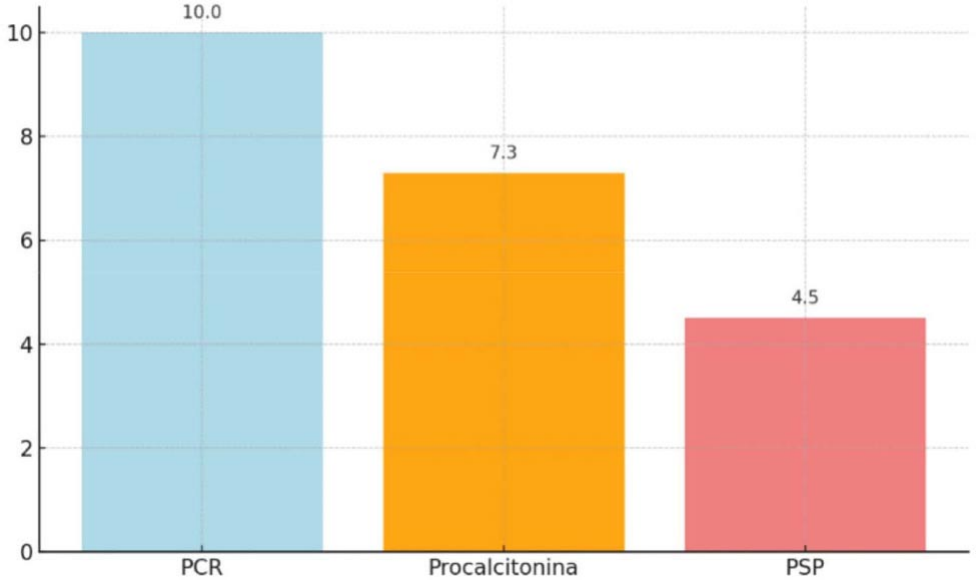
Results of PSP, C-Reactive Protein, and Procalcitonin Levels and Their Association with Length of Stay in the Intensive Care Unit (ICU) of the Hospital Metropolitano de Santiago (HOMS)

**Figure 2.**
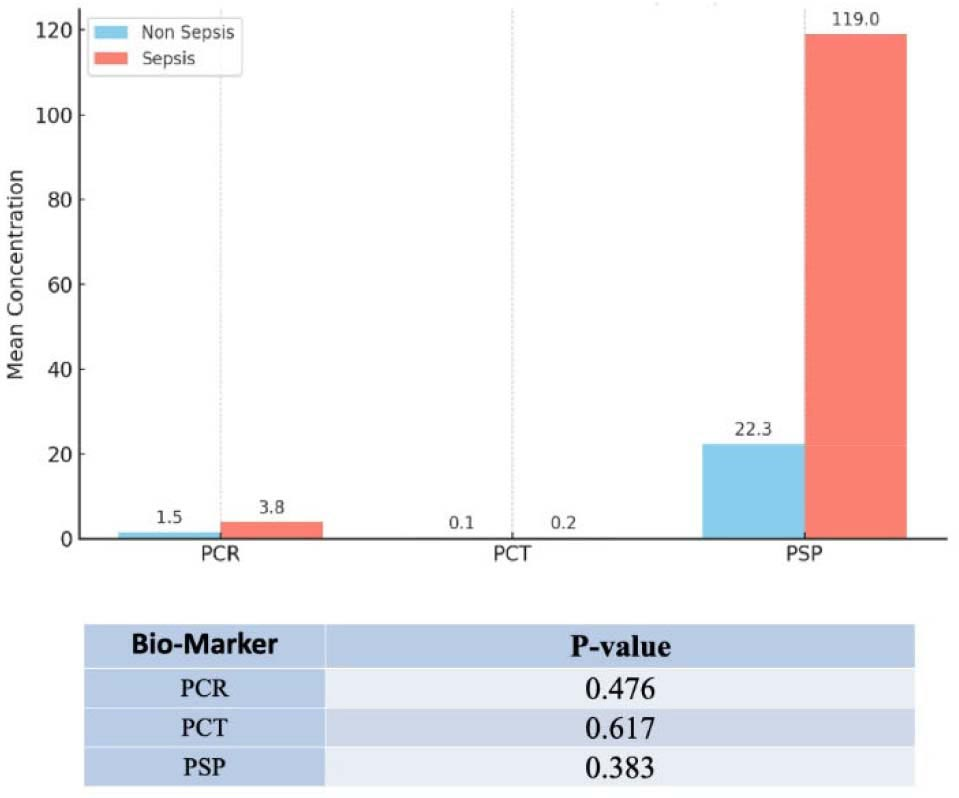
Comparison of Average Levels of PSP, PCR, and PCT and Their Association with Sepsis in Patients Admitted to the Intensive Care Unit (ICU) of the Hospital Metropolitano de Santiago (HOMS)

**Results:**

Among 36 patients, 66.7% were male, with a mean age of 47.2 years. Hypertension was the most common comorbidity (38.9%). PSP levels had a mean of 94.8 ng/mL and a wide range (10–667 ng/mL). CRP and PCT were near zero in most cases, limiting analysis. A strong correlation was found between CRP and PCT (ρ = 0.91, p = 0.0003), but PSP did not correlate with CRP (ρ = 0.15, p = 0.6103) or PCT (ρ = 0.18, p = 0.5382). No statistically significant differences were observed between septic and non-septic patients for any biomarker. Elevated CRP appeared to trend with longer ICU stays (∼10 days). No mortality was recorded during the study period.

**Conclusion:**

CRP and PCT showed strong correlation and may reflect similar inflammatory processes. PSP did not correlate with sepsis or other markers. Further research with larger samples is needed to evaluate PSP’s role in early sepsis diagnosis.

**Disclosures:**

All Authors: No reported disclosures

